# Single-cell multimodal analysis in a case with reduced penetrance of Progranulin-Frontotemporal Dementia

**DOI:** 10.1186/s40478-021-01234-2

**Published:** 2021-08-03

**Authors:** Karthick Natarajan, Jesper Eisfeldt, Maria Hammond, José Miguel Laffita-Mesa, Kalicharan Patra, Behzad Khoshnood, Linn Öijerstedt, Caroline Graff

**Affiliations:** 1 Division for Neurogeriatrics, Department of Neurobiology, Care Sciences and Society, Center for Alzheimer Research, Karolinska Institutet, Stockholm, Sweden; 2 Unit for Hereditary Dementias, Clinical Genetics, Karolinska University Hospital-Solna, Stockholm, Sweden; 3 Department of Molecular Medicine and Surgery, Karolinska Institutet, Stockholm, Sweden; 4 Science for Life Laboratory, Karolinska Institutet Science Park, Solna, Sweden; 5 Department of Immunology, Genetics and Pathology, Science for Life Laboratory, Uppsala University, Uppsala, Sweden

**Keywords:** Progranulin, Haploinsufficiency, Human genetic disorders, Neurodegenerative disorders, Nuclei multiplexing, CITE-Seq, Frontotemporal dementia, Reduced penetrance

## Abstract

We identified an autosomal dominant progranulin mutation carrier without symptoms of dementia in her lifetime (Reduced Penetrance Mutation Carrier, RedPenMC). This resistance to develop expected pathology presents a unique opportunity to interrogate neurodegenerative mechanisms. We performed multimodal single-nuclei analyses of post-mortem frontal cortex from RedPenMC, including transcriptomics and global levels of chromatin marks. RedPenMC had an increased ratio of *GRN*-expressing microglia, higher levels of activating histone mark H3k4me3 in microglia and lower levels of the repressive chromatin marks H3k9me1 and H3k9me3 in the frontal cortex than her affected mutation carrier son and evidence of higher protein levels of progranulin in both plasma and brain homogenates. Although the study is limited to one case, the results support that restoring brain progranulin levels may be sufficient to escape neurodegeneration and FTD. In addition to previously identified modifier genes, it is possible that epigenetic marks may contribute to the increased progranulin expression in cases of reduced penetrance. These findings may stimulate similar follow-up studies and new therapeutic approaches.

**Supplementary Information:**

The online version contains supplementary material available at 10.1186/s40478-021-01234-2.

## Introduction

In 2006, it was demonstrated that pathogenic progranulin (*GRN*) mutations can cause autosomal dominant Frontotemporal dementia (FTD), histopathologically characterised by aggregation of ubiquitin-binding protein p62 and phosphorylated TAR DNA-binding protein 43 (pTDP-43) in the frontal cortex [4, 11, 15]. GRN is a pleiotropic growth factor and pathogenic mutations reduce GRN protein levels in the brain, plasma and cerebrospinal fluid [11]. Various studies identified modifiers of disease risk and age at onset in *GRN* mutation carriers [27, 28, 42, 65, 71]. In rare cases, mutation carriers completely escape the disease phenotype known as “reduced penetrance” [2, 12]. The underlying mechanism behind reduced penetrance is unclear but may be influenced by several factors including transcriptomic and epigenetic modifications as well as other factors [12]. There are distinct changes in the transcriptome and chromatin landscape in different brain regions of dementia when compared to aged controls [5, 7, 26, 58, 69]. Growing evidence is also showing a link between aging and changes in histone profiles, both the number of histone proteins per cell and in the balance of activating and repressing histone modifications [7, 10, 52].

Lately, high-throughput, droplet-based single-nuclei RNA-Seq (snRNA-Seq) has been employed to study different neurological disorders [20, 35, 50, 62]. Moreover, snRNA-Seq has been combined with nuclei hashing using oligo-barcoded antibodies to allow for multiplexing without altering transcriptional profiles [18]. Cellular Indexing of Transcriptomes and Epitopes by Sequencing (CITE-Seq) is a multimodal method that enables simultaneous analysis of transcriptome and protein targets at a single-cell resolution [55].

This study includes a rare case of reduced penetrance (RedPenMC), a 96-year-old female *GRN* mutation (p.Tyr294*) carrier who had no signs of FTD in her lifetime, her affected *GRN* mutation carrier (AMC) son from the same family and an unaffected non-carrier (NC). We used the frontal cortex to perform single-cell multimodal measurements [72] of the transcriptome and global levels of several histone modifications using CITE-Seq [18] since bulk tissue transcriptome analysis will be inadequate to map cell-type-specific molecular changes [62, 72].

### Case presentation

**Fig. 1:**
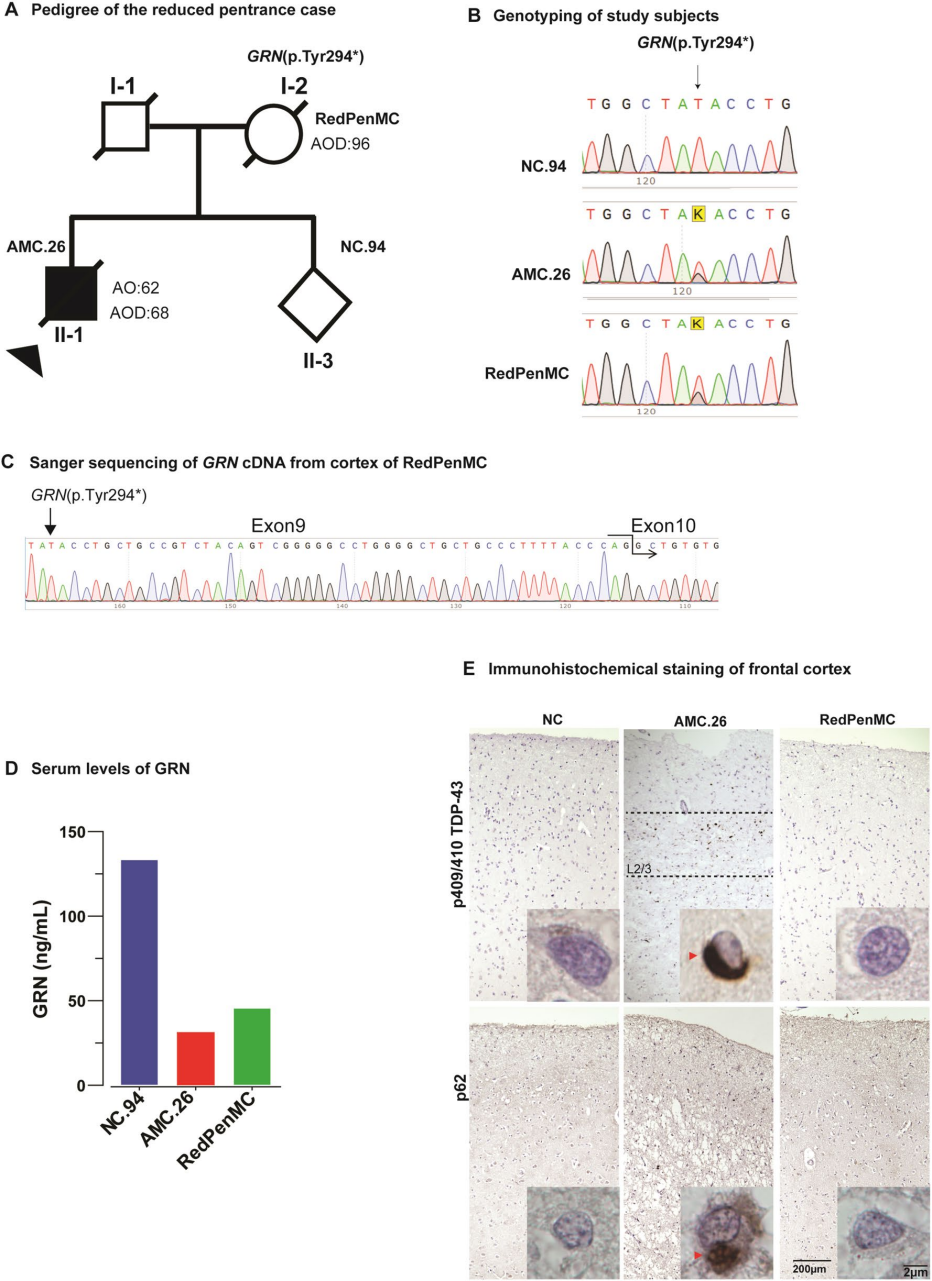
Clinical, genetic and immunohistochemical analysis of AMC and RedPenMC. **A** Pedigree of the RedPenMC (I-2). 96-year-old female with *GRN* (p.Tyr294*) mutation. AOD: age of death, AO: age of symptom onset. Also see Additional file 2: Table S1. **B** Genotyping by Sanger sequencing of the family members for the *GRN* (p.tyr294*) mutation showing the T > G substitution (c.882T > G) at g.42428777T > G known as rs794729670 [11, 39]. **C** Sanger sequencing of GRN (exon9) cDNA generated from frozen brain BA10 of RedPenMC showing only the wild type allele T at position c.882. **D** Serum levels of GRN measured using ELISA in three technical replicates for each donor. **E** Immunohistochemical staining of the frontal cortex of AMC.26 (red arrows) and RedPenMC against p409/410 TDP-43 and p62. A 96-yr-old female NC was used as a control. Hematoxylin was used as a nuclear counterstain. The control paraffin sections were obtained from the Netherlands Brain Bank (NBB, refer Additional file 2: Table S2b). Scale bar:200 µm & 2 µm. L2/3: Cortical layer 2/3

A 96-year-old female (RedPenMC), carrying the pathogenic *GRN* mutation (p.Tyr294*) [11] and who was devoid of any cognitive impairment or neurological abnormalities until death was identified (Fig. 1A, B). Our Sanger sequencing of cDNA (synthesized from total RNA) from frozen prefrontal cortex Brodmann Area 10 (BA10) confirmed that mutant mRNA was not expressed in RedPenMC (Fig. 1C), which is in line with previous data that *GRN* non-sense mutations cause non-sense mediated mRNA decay, leading to haploinsufficiency [53]. The GRN haploinsufficiency of the affected mutation carrier (AMC.26) as well as the RedPenMC, was confirmed by reduced serum GRN levels (Fig. 1D). The Genetic Frontotemporal Dementia Initiative (GENFI) have reported that plasma levels below 61.55 ng/mL is a predictor of GRN mutations [16] and more recently the suggested threshold was 71.0 ng/mL [51]. The relative GRN level was higher in RedPenMC (45.70 ng/ml) compared to AMC.26 (31.49 ng/ml). Whole-genome sequencing (WGS) of blood DNA was used to scan for genetic modifiers associated with *GRN* mediated FTD in RedPenMC, and two of her offspring AMC.26, and the control NC.94 (Additional file 2: Table S5). The genotypes for rs5848, a *GRN* 3’ UTR variant, was (C/T) in AMC.26 and (C/C) in RedPenMC. The ‘C’ allele is a less efficient binding site for miR-659 compared to the ‘T’ allele, where miR-659 is suggested to reduce GRN expression by inhibiting its translation [23]. Another reported modifier known to affect *GRN* levels as well as the age of onset [13, 14, 17, 61] in *GRN* mediated FTD is SNP rs1990622 in the TMEM106B gene, implicated in the proper functioning of the lysosome. The A/G genotype (G protective allele) was present in RedPenMC whereas the A/A genotype (A risk allele) was present in AMC.26. Genotypes of other possible modifier genes (*GFAR2, CDH23, PSAP, CELSR2, SORT1*) are presented in Additional file 2: Table S5. RedPenMC carries only one copy of the rs7869 T-allele associated with increased *PSAP* and lower plasma PGRN levels [37], no C-allele for rs646776 which is associated with increased *SORT1* mRNA levels and reduced plasma PGRN levels [9] and finally RedPenMC is homozygous AA for *GFAR2* where AA homozygotes have an increased disease risk associated with decreased brain *GFRA2* mRNA levels but no reported effect on extracellular levels of PGRN [42].

Immunohistochemistry in RedPenMC showed an absence of immunoreactivity towards p62 and pTDP-43 in the frontal cortex similar to the findings in age and gender-matched NC whereas AMC.26 showed characteristic p62 and pTDP-43 aggregations (Fig. 1E, red arrows).

### Single-cell multimodal analysis of RedPenMC, AMC.26 and NC.38

**Fig. 2:**
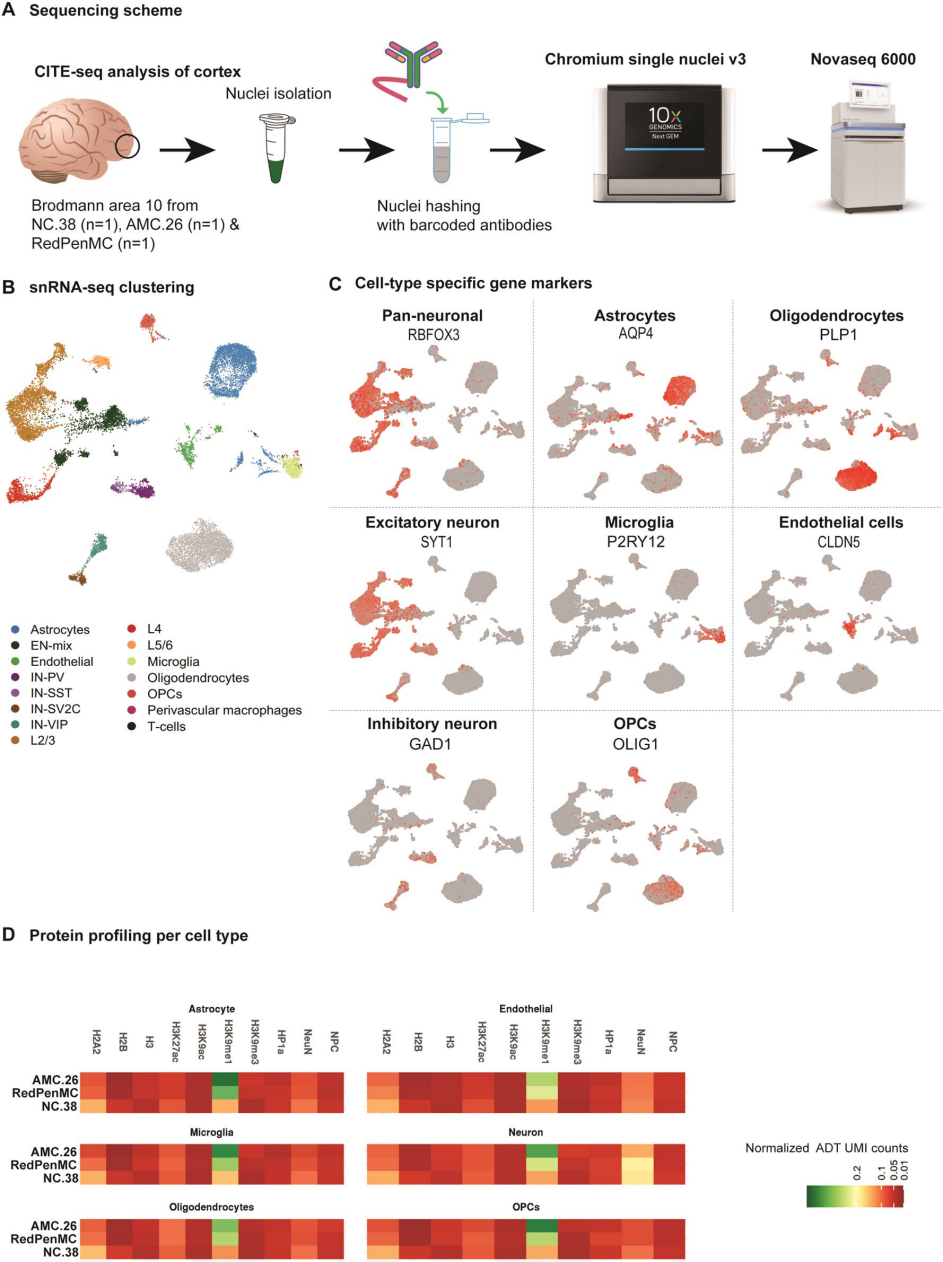
Overview of the experimental approach and CITE-Seq datasets. **A** Cellular Indexing of Transcriptomes and Epitopes by Sequencing (CITE-Seq) multi-modal analysis of nuclei isolated from frozen Brodmann area 10 (BA10) which includes NC.38, AMC.26 and reduced penetrance (RedPenMC, refer Additional file 2: Table S1 & S2a). NC.38 frozen brain was obtained from Mount Sinai Brain bank part of NIH Brain and Tissue Repository (NIH NeuroBioBank, USA). AMC.26 and RedPenMC frozen brain were obtained from Brain Bank at Karolinska Institutet, Sweden. **B** UMAP of integrated snRNA-Seq 18,266 profiles from AMC.26, NC.38 and RedPenMC annotated according to the expression of known markers. **C** Known markers for pan-neuronal, excitatory neurons, glial and endothelial cells (OPCs: Oligodendrocyte progenitor cells). **D** Representative protein profiles for different cell types after normalization of the unique molecular identifier (UMI) counts, (*ADT* Antibody derived tags)

CITE-Seq was performed to comprehend cell-type-specific molecular changes in RedPenMC and AMC.26 (Fig. 2A–D, details refer Additional files 1 & 2). Frozen BA10 from RedPenMC, from an unrelated age and gender-matched control (NC.38) and from AMC.26 (Additional file 2: Table S2a) were processed for unbiased isolation of nuclei and the nuclei were stained for a panel of 15 nuclear proteins (Additional file 2: Table S3) including histone marks followed by sequencing using 10X genomics chromium. After quality control, we obtained 18,266 single nuclear transcriptome profiles from RedPenMC, AMC.26 and NC.38 with a median detection of 2223 expressed genes per cell. In parallel, nuclei hashing yielded 9925 protein profiles after the quality control, we used the Nuclear Pore Complex (NPC) for quantification. Cell clusters were identified using the Seurat single cell analysis toolkit [56], and the distinct cell clusters were annotated according to different cell lineage markers, resulting in the detection of 15 cell types including excitatory and inhibitory cortical neurons, astrocytes and microglia (Fig. 2B, C). Neuronal subtypes included excitatory neurons upper layer *CUX2*, middle layer *RORB*, deep layer *TSHZ2* [59] as well as inhibitory neurons (IN) marked with *GAD1*, which included different subtypes IN-*PV,* IN*-SST,* IN*-VIP* and IN*-SV2C* (Fig. 2C). All the cell types were represented in all three samples except IN-*SST* (only in NC.38), T-cells (only in AMC.26 and RedPenMC) and perivascular macrophages (only in RedPenMC). These cell types were not considered in the subsequent analysis. Using cellular annotation from the transcriptome, we mapped the global levels of 14 nuclear proteins for each cell type (Fig. 2D). The protein panel included chromatin marks: H3k4me3, H3k9me1 and H3k9me3.

**Fig. 3:**
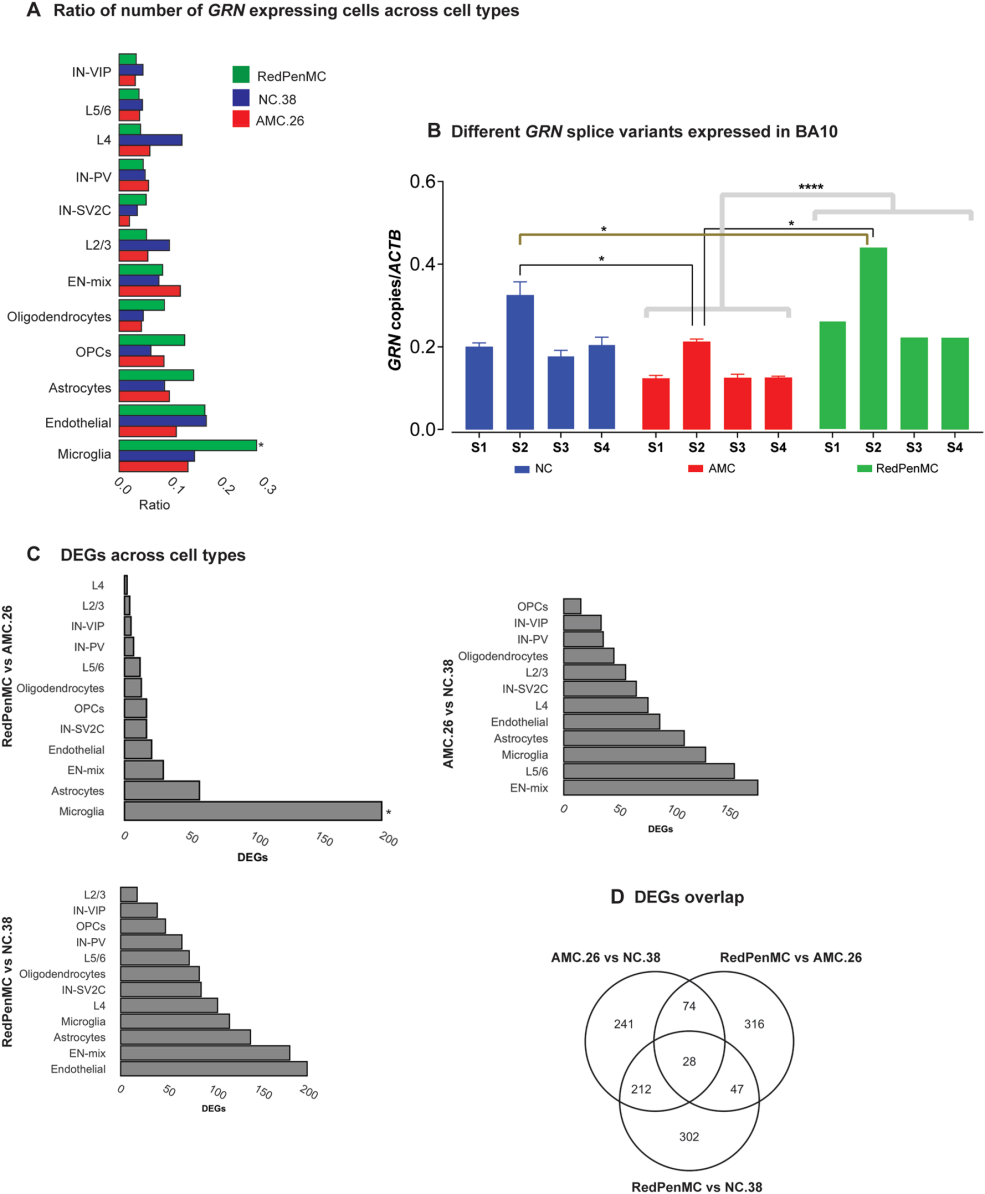
Fraction of *GRN* expressing cells in the BA10 region and cell-type-specific associated changes in RedPenMC and AMC. **A** The ratio between number of *GRN* expressing to non-expressing cells for each cell type in Brodmann area 10 (BA10) sampled from RedPenMC, AMC.26 and NC.38. The P value calculated by comparing different cell types. *indicates *P* < 0.05. **B** Different *GRN* splice variants (S1, S2, S3, S4) expressed in BA10 region of NC (n = 3), AMC (n = 3) and RedPenMC (n = 1) analyzed using Digital Droplet™ PCR (ddPCR). RNA was isolated from the bulk tissue for each donor. *GRN* splice variants were detected amplifying exon-exon junctions, for S1:E1_E2, S2:E13_3’UTR, S3:E6_E7 and S4:E10_E12. Transcript expression is expressed as number of copies per microliter. The threshold was set automatically above any positive signal detected in multiples negative controls that contained a digital droplet PCR (ddPCR) cocktail and water in place of either of the cDNA. Multiple negative controls were included in each plate. Statistical differences in the expression of four splice variants in NC, AMC and RedPenMC were calculated by one-way ANOVA with Bonferroni's multiple comparison (Post Hoc test). *P < 0.05, ****P < 0.005. **C** Differentially expressed genes (DEGs) in RedPenMC, AMC.26 and NC.38 (upregulated and downregulated) obtained from all cell types. *P* values were calculated by comparing the number of DEGs between different cell types per group comparison. *indicates *P* < 0.05. **D** The intersection of DEGs in different comparison groups

We assessed the *GRN* expression by analysing the expression levels of the different *GRN*-splice variants in frozen BA10 (refer Additional file1 & 2) from RedPenMC, AMC (n = 3), and unrelated NC (age and gender-matched to AMC and RedPenMC, n = 3, Fig. 3B). Overall, the total *GRN* expression, including all splice variants (S1, S2, S3, S4) was higher (*P* < 0.005) in RedPenMC compared to AMC. Especially, splice variant ‘S2’ was significantly increased in RedPenMC compared to AMC and NC. The ‘S2’ splice variant upregulated in RedPenMC, includes rs5848 in the 3’ UTR region [44]. These results indicate that total *GRN* expression in bulk RNA was higher in RedPenMC compared to AMC and NC, at least for some isoforms.

GRN is a ubiquitously expressed protein [36]. To characterize the *GRN* expression in BA10 of RedPenMC, we computed the distribution of cells expressing detectable levels of *GRN* across each cell type (Fig. 3A). The fraction of microglia expressing *GRN* was higher in RedPenMC (*P* < 0.05) compared to AMC.26 and NC.38 (Fig. 3A). However, there was only a trend towards a significantly lower total *GRN* expression in AMC.26 compared to RedPenMC (*P* = 0.054, log2fold change − 0.254), possibly due to the low sequencing-depth and relatively low overall signal to noise *GRN* expression levels. We also observed a high concordance between *GRN* expression and *PSAP* expression [71] in microglia in both *GRN* mutation carriers (Additional file 2: Table S4). Western blot analysis of homogenized frozen BA10 showed that RedPenMC had brain progranulin protein-levels closer to the levels in the control NC.38 than the levels in AMC.26 (Additional file 3: Fig. S2A).

To apprehend the transcriptome profile of RedPenMC, we compared nuclear profiles with AMC.26 and NC.38 (Additional file 2: Table S6). We detected 465 differentially expressed genes (DEGs, *P* < 0.05; log2fold change ± 0.5) in the comparison between RedPenMC and AMC.26 (Fig. 3C) and 555 DEGs between AMC.26 and NC.38. Finally, we compared RedPenMC and NC.38, resulting in the detection of 589 DEGs.

In RedPenMC vs AMC.26, the highest number of DEGs were found in microglia followed by astrocytes and endothelial cells. The statistical testing revealed the DEGs burden in microglia were significant (*P* < 0.05) compared to other cell types. Similar DEGs burden analysis per cell type was carried out for AMC.26 vs NC.38 and RedPenMC vs NC.38 and the analysis yielded no significant differences (Fig. 3C). Previously, it was reported that GRN haploinsufficiency can be extended to the microglial expression of *GRN* [20, 49] and loss of *GRN* affects microglial phenotype as well as transcriptome profile. Next, we explored the intersection of DEGs in microglia and 257 Disease-associated microglial genes (DAM) [8, 29, 35, 54] and found *APBB2, PMP22, CD9* to be upregulated in the AMC.26 (Additional file 2: Table S6). GO analysis of DEGs indicated that “astrocyte differentiation”, “oligodendrocyte differentiation” and “regulation of neuron differentiation” were among the enriched dysregulated pathways (Additional file 3: Fig. S1A). Thereafter, we intersected the number of DEGs and GO among three comparison groups described earlier (Fig. 3D, Additional file 3: Fig. S1B). 10% of DEGs were overlapping between RedPenMC vs AMC.26 and AMC.26 vs NC.38. Moreover, 26% of DEGs were overlapping between RedPenMC vs NC.38 and AMC.26 vs NC.38 (Fig. 3D). GO intersection indicates that RedPenMC vs AMC.26 and AMC.26 vs NC.38 share 21% of biological processes (Additional file 3: Fig. S1B). In contrast, GO-intersection analysis of RedPenMC vs NC.38 did not show overlap with any of the other two comparisons (Additional file 3: Fig. S1B). The results may suggest that the identified GO in our study were associated to the pathological phenotype in affected mutation carrier and not the mutation per se or a combination of the mutation and pathological phenotype.

**Fig. 4:**
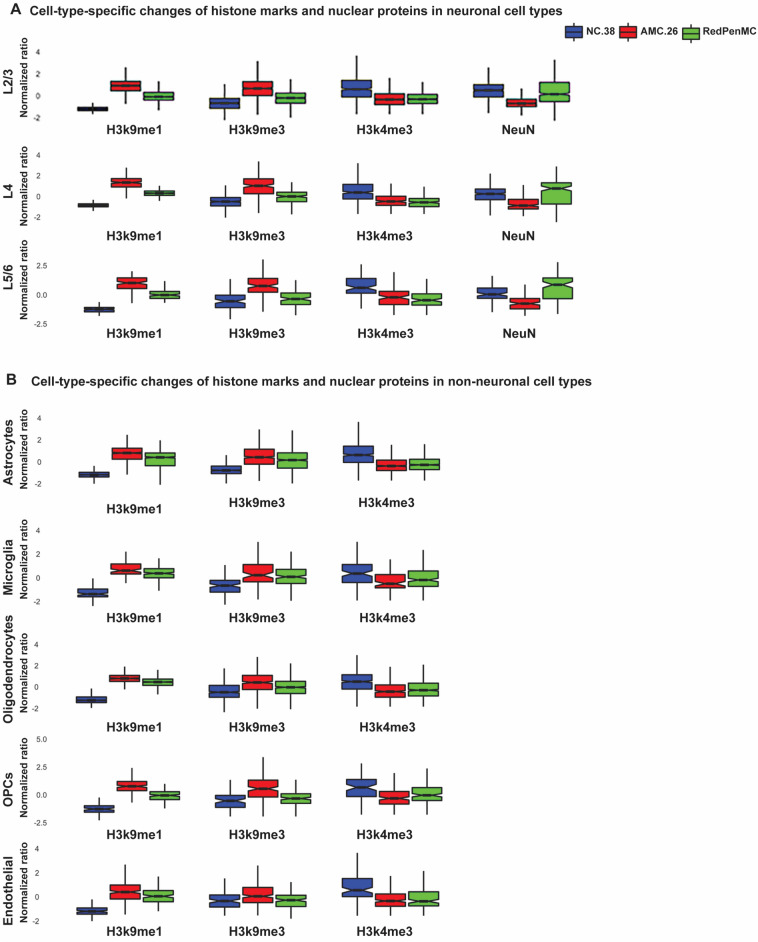
Cell-type-specific changes of histone marks in the BA10 region in RedPenMC and AMC. Global expression profile of 5 distinct nuclear proteins in different cell types among RedPenMC, AMC.26 and NC.38 in neuronal cell types (**A**) and non-neuronal cell types (**B**). The* p* values for the donor versus donor comparison were listed in the Additional file 2: Table S7. The boxplot represent variation among the cells of each cell type in each donor

**Fig. 5:**
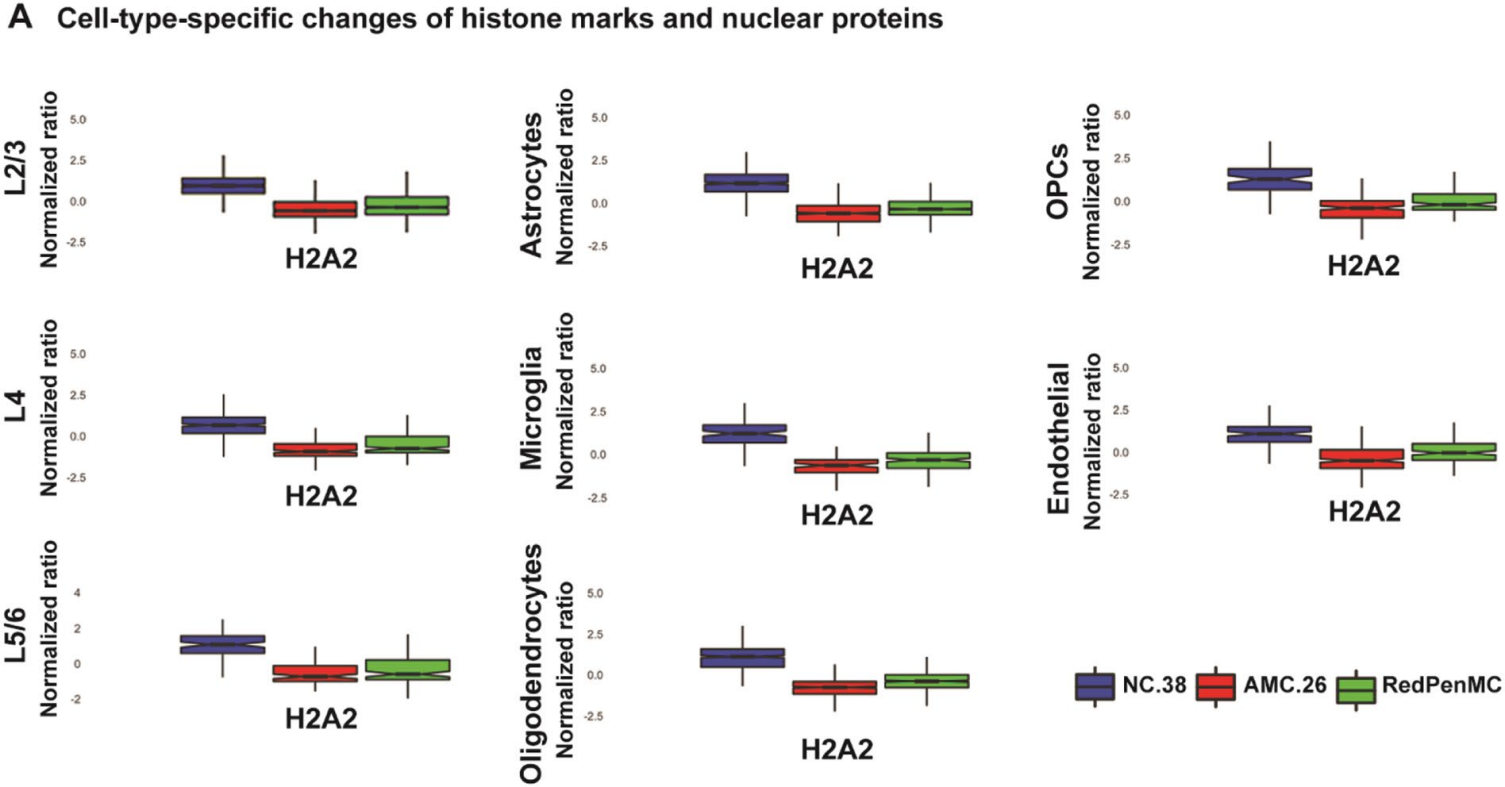
Cell-type-specific changes of H2A2 in the BA10 region in RedPenMC and AMC. **A** Global expression profile of H2A2 in different cell types among RedPenMC, AMC.26 and NC.38. The *p* values for the donor versus donor comparison were listed in the Additional file 2: Table 7. The boxplot represent variation among the cells of each cell type in each donor

Finally, we performed analysis of global changes of open and closed chromatin marks to understand the chromatin state of RedPenMC and AMC.26 across different cell types. These analyses revealed that 9 out of 14 nuclear proteins (Additional file 2: Table S7) were changed (*P* < 0.05) among different cell types in AMC.26, RedPenMC and NC.38 (Fig. 4A, B, 5A).

In cortical layer 2/3 (L2/3), NeuN expression was reduced in AMC.26 compared with RedPenMC and NC.38 (Fig. 4A). Typically, in FTD associated with GRN mutation, cortical layers 2/3 are affected by the loss of neurons [30] and downregulation of NeuN levels [67]. NeuN quantitative reduction in L2/3 is indicative of a decline in neuronal health in the cortex of AMC.26, our results are in agreement with previous findings of FTD mediated by GRN mutations [30, 67].

The repressive mark H3k9me1 and H3k9me3 were overexpressed in AMC.26 compared to RedPenMC and NC.38 across different cell types analysed (Fig. 4A-B). H3k9me1 is a key mark for functional heterochromatin [47, 48] and preferred substrate for suppressor of variegation 3–9 (SUV39) methyltransferase, which catalyses H3k9me3 formation [33, 48]. Elevated levels of H3k9me1 in mouse models of Alzheimer's disease (AD) [63] leads to the downregulation of *BDNF* (brain-derived neurotrophic factor). BDNF is critical for neuronal synaptic plasticity and mediates neuronal differentiation and cognitive functions [40, 46]. Moreover, GRN is co-transported with BDNF within neuronal axon and dendrites [41]. H3k9me3 is associated with transcriptional silencing and altered chromatin plasticity in neurodegeneration [32, 66]. Moreover, H3k9me3 is essential in cell identity establishment and maintenance [36]. Previously, overexpression of H3k9me3 has been reported in AD [32] and Huntington disease (HD) [31]. RedPenMC showed a higher expression of H2A2 than AMC.26 in L2/3, L4, astrocytes, microglia, oligodendrocytes, endothelial cells and OPCs (Fig. 5A). H2A2 is critical in stabilizing differentiated cell identity as well as chromatin compaction [34, 57].

On the other hand, global levels of H3k4me3 were decreased in both RedPenMC and AMC.26 across cell types compared to NC.38 (Fig. 4A, B). However, microglial H3k4me3 expression was higher (*P* < 0.05) in RedPenMC compared to AMC.26. H3k4me3 levels are associated with the upregulation of transcription activity, reflects the extent of transcription and ‘activating’ histones [6, 22]. Recently, Proximity Ligation-Assisted ChIP-Seq (PLAC-Seq) analysis indicated that *GRN* expression is regulated by H3k4me3 in microglia [38]. Previously, reduced levels of H3k4me3 have been reported in HD, and locus-specific loss and gain of H3k4me3 have been implicated in AD [7, 19].

## Conclusions and discussion

Here, we investigated a *GRN* mutation carrier with reduced penetrance using WGS and multi-modal genomics single-cell analyses [49]. Previously, *TMEM106B* [15] and *GFRA2* [42] were identified as modifiers of *GRN* mediated FTD. As of date, we lack a systematic understanding of the disease process and modifiers [65]. Reduced penetrance cases have the potential to uncover endogenous escape mechanisms [2, 12]; providing insights that may be useful for designing future molecular therapies. Naturally occurring cases of reduced penetrance can only be explored using patient-derived materials and cannot be replaced by genome-editing or mouse models [2, 12].

Our bulk ddPCR and Western blot analyses revealed a higher brain progranulin RNA and protein expression in RedPenMC compared to AMC. Our single-cell analyses revealed an increased ratio of *GRN* expressing microglia as well as microglial-specific higher levels of H3k4me3 and lower levels of global H3k9me1 and H3k9me3 expression in RedPenMC compared to AMC. RedPenMC carried one or more extra copies of the “protective” genetic-modifier-variants in *GRN*, *TMEM106B*, *SORT1* and *PSAP* in contrast to her affected son. Although our study is limited by sample size and there is a great need of finding a larger number of RedPenMC for example by collaborative sharing of patient materials or screening genomic population genomic data, such as the SweGen [1], we still think our data can generate some interesting new ideas hypothesis and caveats are presented below.

For GRN haploinsufficiency, the obvious therapeutic approach is to increase the progranulin protein levels. However, it is unclear whether an increase of *GRN* in all cell types, or in specific cell types are needed to maintain normal brain function. Our data suggests that selective increase of *GRN* expression in microglia, with drug modulation [60] or genome engineering [3, 45] may prevent the initiation of the neurodegenerative process which has successfully been demonstrated in mouse models earlier [68]. In addition to that, combinatorial inhibition of G9a, SETDB1 and GLP may contribute to the modulation of histone methylation enzymes; which has been proven effective in models of AD [70] and HD [31]. In addition, the levels of H3k4me3 can be increased by inhibiting the activity of histone deacetylases [25, 64]. However, future studies are needed in RedPenMC to understand the microglial cellular processing of GRN into granulin peptides [24] and its interaction with neuronal and astrocyte counterparts.

The CITE-Seq approach is limited in resolution and by technical artefacts, including doublets and difficulties in assembling the transcripts. These technical limitations could be overcome through cell sorting and running full-transcript length single-cell sequencing [21] on similar materials, allowing the pinpointing of specific transcripts (alternative splice forms) in different cell populations. Alternatively, microglia could be sorted from BA10 and then sequenced using long-read sequencing. Genome-wide single-cell chromatin accessibility [43] analysis of BA10 is needed to understand cell-type-specific H3k9me3 and H3k9me1 binding sites in RedPenMC, AMC and NC. Although the two CITE-sequenced mutation carriers included were biological relatives and obtained via the same brain biobank, the control was obtained from another biobank, was unrelated and matched with respect to age and sex only to RedPenMC which may further limit the interpretation of the data.

To conclude, our preliminary analysis of a single reduced penetrance case indicates that the presence of sustainable levels of both RNA and protein progranulin, at least in some cell types in brain, may be the result of both genetic as well as epigenetic modifiers. It is possible that a near normal level of progranulin is sufficient to preserve cognition and prevent neurodegeneration but needs to be further explored and we hope our data can generate new testable hypothesis for therapeutic intervention in presymptomatic *GRN* carriers.

## Supplementary Information

**Additional file 1**. Supplementary methods and materials.

**Additional file 2**. Supplementary tables.

**Additional file 3**. Supplementary figures.

## Data Availability

CITE-Seq datasets are available at the ArrayExpress (https://www.ebi.ac.uk/arrayexpress/) with accession ID: E-MTAB-9530.
