# Supplementary material for: Single-cell multimodal analysis in a case with reduced penetrance of Progranulin-Frontotemporal Dementia
Source: Acta Neuropathol Commun. 2021 Aug 3;9:132. doi: 10.1186/s40478-021-01234-2 (PMC8336016; doi:10.1186/s40478-021-01234-2)
Supplement: Supplementary file 1 — Additional file 1. Supplementary methods and materials. [file 40478_2021_1234_MOESM1_ESM.docx]

**Supplementary methods and materials**

**Study subjects**

The study subjects in Figure 1A (Supplementary Table 1) were part of the familial Frontotemporal Dementia (FTD) study carried out at Karolinska Institutet and recruited through the Unit for hereditary dementias, Karolinska University Hospital, Stockholm as described before [3]. An autosomal dominant pathogenic *GRN* mutation p.Tyr294* (exon9) was identified in our Swedish FTD cohort. After informed consent, research materials were collected from study subjects. All the study subjects, different sample types included as well as different datasets generated from respective sample collections are listed elsewhere (Supplementary Table 1, 2a, 2b). All the procedures were approved by the local ethics committee, Stockholm, Sweden.

**Human brain samples**

Brain samples and the metadata used in this study are listed in Supplementary Tables 2a-b. Frozen prefrontal cortex (Brodmann Area, BA10) samples from AMC and a RedPenMC, archived at the Brain Bank at Karolinska Institutet, Stockholm (https://ki.se/en/nvs/the-brain-bank-at-karolinska-institutet), were used for this study (Supplementary Table 2a). The age and gender-matched frozen BA10 from NC cases were procured from NIH NeuroBioBank, USA (https://neurobiobank.nih.gov/). For immunohistochemistry (Supplementary Table 2b), AMC and RedPenMC formalin fixed paraffin brain sections (FFPE) were provided from the Brain Bank at KI and NC were procured from Netherlands Brain Bank (NBB) (https://www.brainbank.nl/).

**Immunohistochemistry**

Five micrometer thick sections were collected from formalin fixed paraffin embedded tissue from the human frontal cortex and baked at 60 °C for 60 min followed by deparaffinization, with gradual rehydration. Heat-induced antigen retrieval was carried out with incubation at 110 °C for 30 min in a decloaking chamber (Biocare Medical, USA) with either Diva decloaker (Biocare Medical, USA) for antibodies detecting p409/410 TDP-43 or distilled water for p62. Tissue sections were further treated with peroxidase blocker for 15 min (Dako, USA) and unspecific antibody blocking buffer for 60 min (Dako, USA). Primary antibodies against p409/410 TDP43 (1:20000, Cosmo Bio), and p62 (1:1000, Enzo Lifesciences) were diluted in antibody diluent (Dako) at ambient temperature for 60 min. Following washes in TBS buffer, proteins were visualized by 3,3´-diaminobezidine based EnVision kit (Dako). High-resolution images were captured using a Nikon Eclipse E800 bright field microscope.

**Genotyping *GRN* mutation and *GRN* modifiers**

DNA was extracted from fibroblasts or venous blood using the Gentra Puregene Blood Kit (Qiagen, Germany) as per the recommended protocol by the manufacturer. Then 20ng of the isolated DNA was amplified using primers for *GRN*-exon2 & exon9 using AmpliTaq Gold® 360 PCR Master Mix (Thermofisher, USA). The Sanger sequencing was performed using the Big Dye® Terminator v3.1 Cycle Sequencing Kit (Thermofisher, USA). The sequences were analyzed using ABI3500 Genetic Analyzer (Thermofisher, USA). Based on literature evidence, various genetic modifiers of *GRN* were extracted from WGS data (see below) and confirmed using Sanger sequencing. The primer pairs can be available on request.

**Whole-Genome Sequencing (WGS) and variant analysis**

2-2.5µg of blood-derived DNA from NC.94, AMC.26 and the RedPenMC was sequenced using a 30X PCR free protocol at Scilifelab, Stockholm (https://ngisweden.scilifelab.se/), Sweden as per accredited protocols. The variant calling was performed using an established GATK pipeline (Broad Institute, USA) as described before [4, 5]. The .vcf files were imported into the Ingenuity Variant Analysis (Qiagen bioinformatics, Germany) to extract the single nucleotides variation (SNV) of the respective genes.

**Total RNA isolation**

Frozen frontal cortex (50-100mg, Supplementary Table 2a) was lysed using TRIzol™ Reagent (Thermofisher, USA). Lysed samples were purified using the RNeasy Mini Kit (Qiagen, Germany). The purity of the isolated RNA was examined using Agilent 2100 BioAnalyzer (Agilent Technologies, USA).

**Droplet Digital™ PCR**

MIQE guidelines [1] were followed for developing and data analysis of droplet digital PCR. cDNA was synthesized using SuperScript™ VILO™ Master Mix (Thermofisher, USA). Three microliters of cDNA per sample were mixed with QX200™ ddPCR™ EvaGreen® Supermix (Bio-Rad, Hercules, USA) with conditions kept according to the manufacturer’s instructions. Two replicates per sample were run. Moreover, three different genes were used for controlling cDNA input: *ATCB*, *RPL13*, *UBE2D2* but after several trials, the two first were most stable. The pre-PCR mixture was emulsified in 1nL droplets yielding 20000 droplets per sample in the automatic QX100 Droplet generator (Bio-Rad). The PCR reaction mix contained (20μl) and was emulsified with 70μl of QX200™ droplet generation oil for EvaGreen® per well (Bio-Rad). PCR reactions were performed in a Bio-Rad T100 Thermal Cycler with annealing temperature set at 56.6ºC, and ramp rate set down at 2°C. Droplet fluorescence was measured using a QX200 droplet reader (Bio-Rad) and data was analyzed using the QuantaSoft™ Analysis Pro (Bio-Rad). The threshold was set automatically against multiple negative controls consisting of non-template (H2O) PCR reactions. Primers were designed based on the *GRN* gene code sequence: ENST00000053867.8, and for the two splice variants the GENCODE Transcript: ENST00000589265.5 and ENST00000639447.1 (GRCh38/hg38 assembly). The sequence for all gene primers are available on request.

**Nuclei isolation from frozen brain samples**

All the procedures were carried out on ice or ice-cold conditions. Frozen brain tissue (~300 mg) was transferred to a dounce homogenizer (pre-chilled) containing 1 ml of ice-cold Nuclei ez lysis buffer (Sigma Aldrich, USA) supplemented with 1µl DAPI (5mg/ml), 1X protease inhibitor and 0.4U/ml RNase inhibitor. Tissue was lysed gently with 20 strokes with loose ‘pestle A’ and then with 20 strokes with ‘pestle B’ (minimizing bubble formation). Dounce was washed with 1 mL lysis buffer 5 times to recover residual nuclei and incubated 5 min on ice to ensure complete lysis of cell membranes. The nuclei were pelleted by centrifugation 500g, 5 min at 4°C, and the pellet was resuspended in 1ml of ez lysis buffer to count the nuclei using Countess FL II with a DAPI filter. For each vial, 1.25 million nuclei were frozen using the nuclei storage buffer (NSB; Sigma Aldrich, USA).

**Oligonucleotide-conjugation with antibodies (CITE-Seq probes)**

The antibodies were conjugated to azide-modified DNA oligonucleotides (Supplementary Table 3) using DBCO-NHS ester cross-linker (Sigma Aldrich, USA) as previously described [17], using cross-linker: antibody ratio 30:1 and oligonucleotide: antibody ratio 3.33:1. For successful conjugation, the antibodies were at a concentration >1 µg/µl in pure PBS without any NaN3 or primary amines, so if necessary the antibodies were buffer exchanged to PBS using 7 MWCO Zeba columns (Thermofisher, USA) and concentrated using Amicon 30kDa spin columns (Sigma Aldrich, USA). BSA was removed using the Melon Gel IgG Spin Purification kit (Thermofisher, USA). After confirming successful conjugation by polyacrylamide gel electrophoresis, NaN3 (0.05% final concentration) was added to conjugates to quench further conjugation. Antibody-DNA conjugates were pooled at equal ratios. Unconjugated oligonucleotides were removed from the pooled conjugates using Amicon 100kDa spin columns (Sigma Aldrich, USA).

**Nuclei staining**

Nuclear protein staining was performed as described before [12] with few modifications. All the steps were carried out in cold conditions or on ice unless stated otherwise. For nuclei multiplexing, frozen nuclei were thawed in the NSB buffer supplemented with PBS, 2% BSA, 0.02% Tween20, 0.2U/ul RNase Inhibitor and proteinase inhibitor (1x) and spun at 500g for 5 min. The supernatant was discarded and the nuclei resuspended in the NSB buffer and filtered through the 50µm strainer. The nuclei suspension (1 million nuclei) was blocked with 5µl of human TruStain FcX™ (Biolegend, USA) for 10 min on ice. One µg of each of the oligonucleotide-conjugated antibody (Supplementary Table 3) was used for staining each sample. The conjugated antibodies were validated for nuclear localization using confocal microscopy. Briefly, the primary antibody staining was performed with 60 min incubation followed by incubation for 30 min in a tube rotator with an Alexa Fluor®-conjugated secondary antibody (Thermofisher, USA). The nuclear localization of the protein targets was examined using LSM 780 confocal microscopy (Carl Zeiss AG, Germany) along with isotype controls and non-primary antibody controls. All confocal images were processed with ImageJ software [14].

**CITE-Seq**

Samples (NC.38, AMC.26 and RedPenMC) were thawed and processed simultaneously. The antibody staining of nuclei was performed in the cold room as described above. Non-hashed nuclei from the same individuals were then pooled before counting. This allowed us to investigate potential effects on the cDNA library of binding antibodies to histone proteins, background signals from unbound antibodies and the number of nuclei doublets. Then the nuclei were counted and adjusted to 1000 nuclei/µl and submitted for library preparation at the SNP&SEQ Technology Platform, Uppsala, Sweden (http://snpseq.medsci.uu.se/) using 10x Chromium Single Cell 3' Solution v3 as per manufacturer’s protocol. The antibody derived libraries were pooled with the cDNA libraries in a 1:10 ratio. Sequencing was performed on two lanes of a NovaSeq SP flow cell, so at least 650 million read pairs (28+8+0+91) are expected, or on average at least 43000 reads per nucleus. The graphical icons were obtained either from the Reactome Pathway Database icon library (https://reactome.org/icon-lib) or the labicons (<http://www.labicons.net>). 10X genomics instrument image was provided by 10X genomics. NovaSeq6000 instrument image was provided by illumina.

**External datasets used**

Cell type markers for the single nuclei data-analyses were obtained from previously published studies [9, 13, 16].

**CITE-Seq data analysis**

- **snRNA-Seq data analysis**

The resulting single nuclei transcriptome and antibody data (NC.38, AMC.26 and RedPenMC) was pre-processed and aligned to GRCh37 using Cell Rangerv3.1 (https://support.10xgenomics.com/single-cell-gene-expression). We used a pre-mRNA reference transcriptome annotation, which was prepared according to (<https://support.10xgenomics.com/single-cell-gene-expression/software/pipelines/latest/advanced/references>). Next, the transcriptome data was analyzed according to the Seurat 3.1.1 standard practices as described in (https://satijalab.org/seurat/vignettes.html), using the filtered Cell Ranger count matrices as input. Droplets were filtered based on the maximum percentage of mitochondrial Unique Molecular Identifier (UMI) (20%), and minimum feature count (1000 features). Next, doublets were removed using DoubletFinder 2.0.2 [11]. Additionally, we removed mitochondrial genes, postmortem interval associated genes [18], as well as genes known to having a strong gender-biased expression [8], as such genes may affect the downstream analyses. Each of the three quality-controlled transcriptome datasets were clustered and annotated separately. Clusters were generated using the Seurat 3.1.1[15] ‘FindClusters’ algorithm, and differential expression analysis was performed using the MAST1.12.0 [6]. The cell type identity of these clusters were predicted using previously published cell-type-specific markers; initially, each cluster was annotated using a greedy algorithm that searches for the cell type having the largest number of cell-type-specific markers overlapping the 30 most upregulated genes, next the cell type annotation was manually assessed and fine-tuned. Unclear clusters appearing as a mixture of cell types were subclustered and relabeled iteratively.

- The three donors were integrated into a single UMAP using the Seurat package; briefly, the count matrices of each donor were scaled using the ‘ScaleData’ function. The scale data function was run using a set of previously published cell-type-specific markers [13, 16]. Next integration anchors were found using the Seurat ‘FindIntegrationAnchors’ function, lastly, the dataset was integrated running the ‘IntegrateData’ function, and the results were visualized using a UMAP plot.
- The number of expressed genes per cell was computed by counting the number of genes per cell having non-zero expression; and differences regarding the fraction of cells expressing *GRN* was analyzed using binomial tests.
- **Single nuclei Antibody Derived Tags (ADT) analysis**

The antibody counts were preprocessed separately from the transcriptome count data. Empty droplets were separated from cells by finding the so-called “elbow point” [10] in the cumulative UMI antibody count per cellular barcode distribution plot. Next, the background noise level for each antibody was assessed using the Nuclear Pore Complex (NPC) only cells, using an approach similar to [7] and the cell type of the remaining cells was determined based on the snRNA-seq analysis. We excluded all the interneuron subtypes due to the low number of protein profiles per subtype. Lastly, the antibody UMI counts were normalized and visualized as done in previous studies [2]; using standard normal variate (SNV) scaling and the statistical significance of any change was assessed through donor vs donor t-test (P<0.05).

**Measurement of GRN by ELISA**

GRN levels in serum were measured using Progranulin (human) ELISA Kit from AdipoGen Lifesciences (San Diego, USA) as described by the manufacturer. Frozen serum was thawed on ice, 100µl was used for the ELISA at 1:200 dilution. The concentration of GRN in the samples was interpolated from a standard curve using GraphPad Prism v8.3.0.

**Western blot of homogenized frozen brain tissue**

Frozen brain tissue (BA10) was homogenized using Dounce pestles in ice cold RIPA lysis buffer (Thermofisher, USA) complemented with Complete protease inhibitor cocktail (Roche, Switzerland) and PhosSTOP™ (Roche, Switzerland). The lysate where then cleared by centrifugation at 20000 g in 4˚C. BCA protein Assay kit (Thermofisher, USA) was used for protein concentration measurement. Proteins extracts were mixed with 4X Laemmli Sample Buffer (Bio-rad, USA) complemented with 10% 2-Mercaptoethanol, heated in 95˚C used for western blotting using 4–15% Mini-PROTEAN® TGX™ protein gels (Bio-rad, USA). The proteins were transferred on 0.2µm PVDF membrane (Bio-rad, USA) and were blocked in 5% BSA. Blotting was done using Anti-α-Tubulin antibody (1:4000, Merck, USA) and Human Progranulin/PGRN Antibody (1:500, R&D Systems, USA, AF-2420), overnight in 4˚C. Secondary antibodies IRDye® 800-Mouse (1:10000, LI-COR, USA) and HRP-Goat (1:3000, Abcam, UK) were used and the membrane was developed using LI-COR odyssey system. Signal intensity of the bands were measured using ImageJ [14].

**Statistical analysis**

All the statistical analyses were performed using GraphPad prism v8.3.0 unless stated otherwise. No statistical analysis was performed to predetermine the sample size. IHC analysis was performed without prior knowledge of the mutation status. Experiments and data collection were not randomized, NC, AMC and RedPenMC analyses were performed in parallel.

**Legends for Supplementary tables**

**Supplementary table 1**

List of study participants in Figure 1A in this study, sample details and datasets generated in this study.

**Supplementary table 2a**

List of frozen brain samples and their metadata used in this study.

**Supplementary table 2b**

List of paraffin brain sections and their metadata used in this study.

**Supplementary table 3**

List of CITE-Seq probes used in this study.

**Supplementary table 4**

Analysis of *GRN* and *PSAP* expression in neuron and microglia. Shows the ratio of cells expressing *GRN(GRN*_ratio) and *PSAP(PSAP*_Ratio) as week as the ratio of *GRN* expressing cells that also express *PSAP(PSAP_GRN*_ratio).

**Supplementary table 5**

List of GRN mediated FTD associated modifiers and gene variants. Respective genetic variants present in RedPenMC, AMC.26 and NC.94 were extracted from Whole-Genome-Sequencing data using Ingenuity Variant Analysis.

**Supplementary table 6**

Differentially expressed genes (DEGs) between RedPenMC, AMC.26 and NC.38 for each cell type.

**Supplementary table 7**

Differentially expressed proteins between RedPenMC, AMC.26 and NC.38 for each cell type.

**References:**

1 Bustin SA, Benes V, Garson JA, Hellemans J, Huggett J, Kubista M, Mueller R, Nolan T, Pfaffl MW, Shipley GLet al (2009) The MIQE guidelines: minimum information for publication of quantitative real-time PCR experiments. Clin Chem 55: 611-622 Doi 10.1373/clinchem.2008.112797

2 Cheung P, Vallania F, Warsinske HC, Donato M, Schaffert S, Chang SE, Dvorak M, Dekker CL, Davis MM, Utz PJet al (2018) Single-Cell Chromatin Modification Profiling Reveals Increased Epigenetic Variations with Aging. Cell 173: 1385-1397.e1314 Doi 10.1016/j.cell.2018.03.079

3 Chiang HH, Forsell C, Lilius L, Oijerstedt L, Thordardottir S, Shanmugarajan K, Westerlund M, Nennesmo I, Thonberg H, Graff C (2013) Novel progranulin mutations with reduced serum-progranulin levels in frontotemporal lobar degeneration. Eur J Hum Genet 21: 1260-1265 Doi 10.1038/ejhg.2013.37

4 Eisfeldt J, Martensson G, Ameur A, Nilsson D, Lindstrand A (2019) Discovery of Novel Sequences in 1,000 Swedish Genomes. Molecular biology and evolution: Doi 10.1093/molbev/msz176

5 Eisfeldt J, Pettersson M, Vezzi F, Wincent J, Kaller M, Gruselius J, Nilsson D, Syk Lundberg E, Carvalho CMB, Lindstrand A (2019) Comprehensive structural variation genome map of individuals carrying complex chromosomal rearrangements. Plos Genet 15: e1007858 Doi 10.1371/journal.pgen.1007858

6 Finak G, McDavid A, Yajima M, Deng J, Gersuk V, Shalek AK, Slichter CK, Miller HW, McElrath MJ, Prlic Met al (2015) MAST: a flexible statistical framework for assessing transcriptional changes and characterizing heterogeneity in single-cell RNA sequencing data. Genome Biol 16: 278 Doi 10.1186/s13059-015-0844-5

7 Gaublomme JT, Li B, McCabe C, Knecht A, Yang YM, Drokhlyansky E, Van Wittenberghe N, Waldman J, Dionne D, Nguyen Let al (2019) Nuclei multiplexing with barcoded antibodies for single-nucleus genomics. Nature communications 10: Doi ARTN 2907

10.1038/s41467-019-10756-2

8 Grubman A, Chew G, Ouyang JF, Sun G, Choo XY, McLean C, Simmons RK, Buckberry S, Vargas-Landin DB, Poppe Det al (2019) A single-cell atlas of entorhinal cortex from individuals with Alzheimer’s disease reveals cell-type-specific gene expression regulation. Nat Neurosci 22: 2087-2097 Doi 10.1038/s41593-019-0539-4

9 Lake BB, Ai R, Kaeser GE, Salathia NS, Yung YC, Liu R, Wildberg A, Gao D, Fung HL, Chen Set al (2016) Neuronal subtypes and diversity revealed by single-nucleus RNA sequencing of the human brain. Science 352: 1586-1590 Doi 10.1126/science.aaf1204

10 Luecken MD, Theis FJ (2019) Current best practices in single-cell RNA-seq analysis: a tutorial. Mol Syst Biol 15: e8746 Doi 10.15252/msb.20188746

11 McGinnis CS, Murrow LM, Gartner ZJ (2019) DoubletFinder: Doublet Detection in Single-Cell RNA Sequencing Data Using Artificial Nearest Neighbors. Cell Systems 8: 329-+ Doi 10.1016/j.cels.2019.03.003

12 Sardo L, Lin A, Khakhina S, Beckman L, Ricon L, Elbezanti W, Jaison T, Vishwasrao H, Shroff H, Janetopoulos Cet al (2017) Real-time visualization of chromatin modification in isolated nuclei. J Cell Sci 130: 2926-2940 Doi 10.1242/jcs.205823

13 Schirmer L, Velmeshev D, Holmqvist S, Kaufmann M, Werneburg S, Jung D, Vistnes S, Stockley JH, Young A, Steindel Met al (2019) Neuronal vulnerability and multilineage diversity in multiple sclerosis. Nature 573: 75-82 Doi 10.1038/s41586-019-1404-z

14 Schneider CA, Rasband WS, Eliceiri KW (2012) NIH Image to ImageJ: 25 years of image analysis. Nat Methods 9: 671-675 Doi 10.1038/nmeth.2089

15 Stuart T, Butler A, Hoffman P, Hafemeister C, Papalexi E, Mauck WM, 3rd, Hao Y, Stoeckius M, Smibert P, Satija R (2019) Comprehensive Integration of Single-Cell Data. Cell 177: 1888-1902.e1821 Doi 10.1016/j.cell.2019.05.031

16 Velmeshev D, Schirmer L, Jung D, Haeussler M, Perez Y, Mayer S, Bhaduri A, Goyal N, Rowitch DH, Kriegstein AR (2019) Single-cell genomics identifies cell type–specific molecular changes in autism. 364: 685-689 Doi 10.1126/science.aav8130 %J Science

17 Yan J, Gu GJ, Jost C, Hammond M, Plückthun A, Landegren U, Kamali-Moghaddam M (2014) A universal approach to prepare reagents for DNA-assisted protein analysis. Plos One 9: e108061 Doi 10.1371/journal.pone.0108061

18 Zhu Y, Wang L, Yin Y, Yang E (2017) Systematic analysis of gene expression patterns associated with postmortem interval in human tissues. Sci Rep 7: 5435 Doi 10.1038/s41598-017-05882-0
