# Supplementary material for: Single-cell multimodal analysis in a case with reduced penetrance of Progranulin-Frontotemporal Dementia
Source: Acta Neuropathol Commun. 2021 Aug 3;9:132. doi: 10.1186/s40478-021-01234-2 (PMC8336016; doi:10.1186/s40478-021-01234-2)
Supplement: Supplementary file 3 — Additional file 3. Supplementary figures. [file 40478_2021_1234_MOESM3_ESM.docx]

**
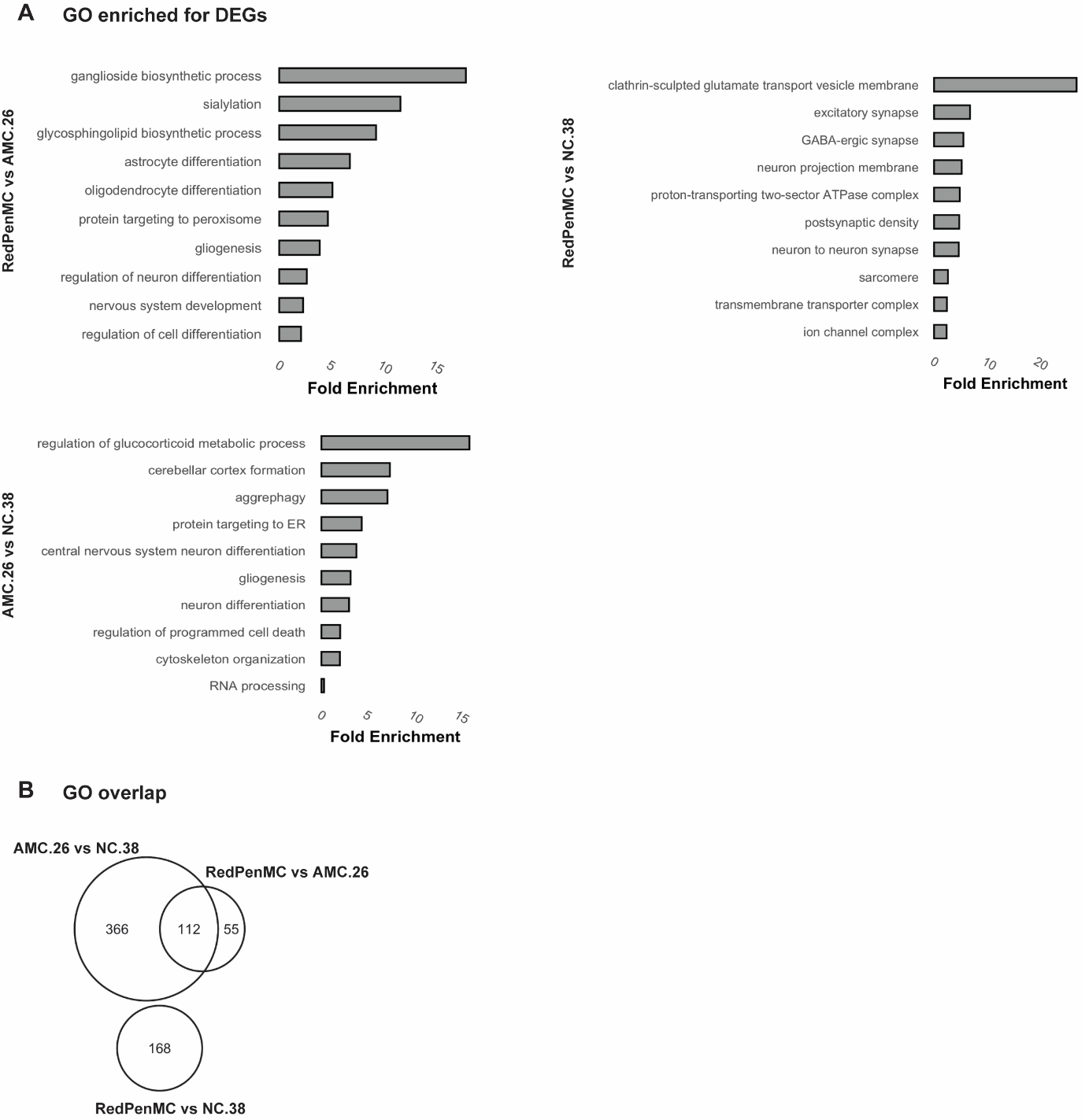
**

**Supplementary figure S1 Gene ontology analysis of differentially expressed genes**

A) Gene ontologies (GO) enriched for DEGs.

B) The intersection of GO in different comparison groups.


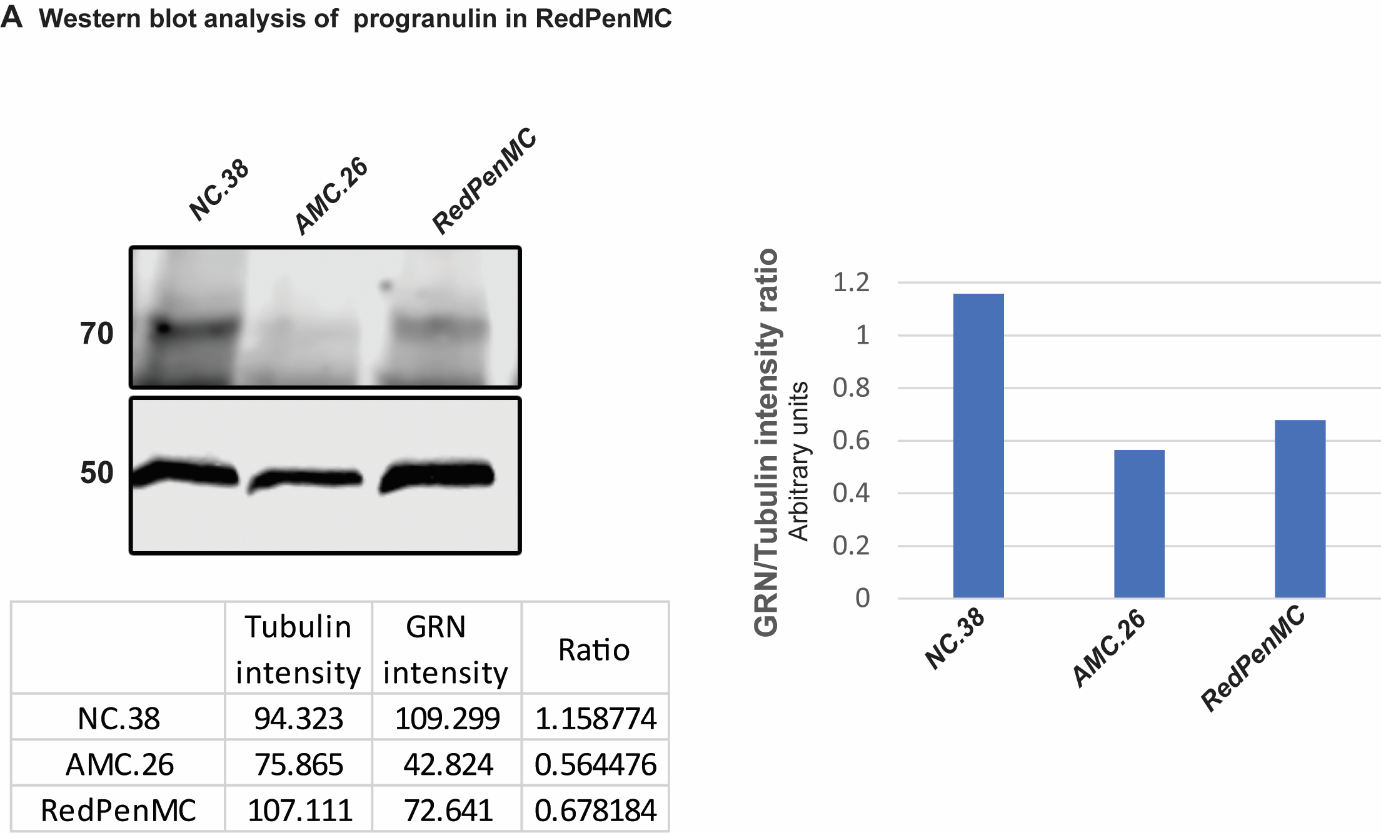


**Supplementary figure S2 GRN analysis in frontal cortex of RedPenMC**

A) Western blot analysis of GRN protein expression in brain lysates (frontal cortex, BA10) from an affected mutation carrier (AMC.26) compared with reduced penetrance case (RedPenMC) and a control sample (NC.38). The signal intensity quantification of the bands suggests a decreased expression of GRN in AMC.26 relative to control, a decrease in RedPenMC relative to the control but a marginal increase in expression of GRN in the RedPenMC relative to AMC.26. WB was repeated independently two different times.
